# Population diversity and multiplicity of infection in *Theileria annulata*

**DOI:** 10.1016/j.ijpara.2010.08.004

**Published:** 2011-02

**Authors:** William Weir, Tülin Karagenç, Mohamed Gharbi, Martin Simuunza, Suleyman Aypak, Nuran Aysul, Mohamed Aziz Darghouth, Brian Shiels, Andrew Tait

**Affiliations:** a University of Glasgow, College of Medical, Veterinary and Life Sciences, Garscube Campus, Bearsden Road, Glasgow G61 1QH, UK; b Adnan Menderes University, Faculty of Veterinary Medicine, Department of Parazitoloji, Batı Kampus, Işıklı, Aydın, Turkey; c Ecole Nationale de Médecine Vétérinaire de Sidi Thabet, Department of Clinical Sciences, Laboratory of Parasitology, 2020 Sidi Thabet, Tunisia

**Keywords:** *Theileria annulata*, Population genetics, Micro-satellites, Mini-satellites, Genetic exchange, Multiplicity of infection, Vaccination

## Abstract

The tick-borne apicomplexan parasite *Theileria annulata* is endemic in many sub-tropical countries and causes the bovine disease tropical theileriosis. Although the parasite is known to be highly diverse, detailed information is lacking on the genetic structure of natural populations and levels of multiplicity of infection in the cattle host. With the widespread deployment of live attenuated vaccines and the emergence of drug-resistant parasites in the field, it is vital to appreciate the factors which shape genetic diversity of the parasite both within individual hosts and in the wider population. This study addresses these issues and represents an extensive genetic analysis of *T. annulata* populations in two endemic countries utilising a high-throughput adaptation of a micro- and mini-satellite genotyping system. Parasite material was collected from infected cattle in defined regions of Turkey and Tunisia to allow a variety of analyses to be conducted. All animals (*n* = 305) were found to harbour multiple parasite genotypes and only two isolates shared an identical predominant multi-locus profile. A modelling approach was used to demonstrate that host age, location and vaccination status play a measurable role in determining multiplicity of infection in an individual animal. Age was shown to positively correlate with multiplicity of infection and while positive vaccination status exerted a similar effect, it was shown to be due not simply to the presence of the immunising genotype. Importantly, no direct evidence was found for the immunising genotype spreading or recombining within the local parasite community. Genetic analysis confirmed the tentative conclusion of a previous study that the parasite population appears to be, in general, panmictic. Nevertheless, evidence supporting linkage disequilibrium and a departure from panmixia was uncovered in some localities and a number of explanations for these findings are advanced.

## Graphical abstract

**Graphical abstract:**

**Figure:**
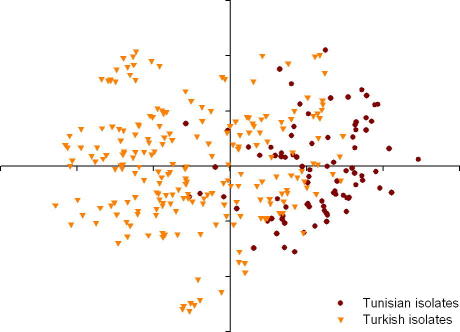

**Research highlights:**

► Natural populations of *Theileria annulata* show extensive diversity. ► Evidence of panmixia with linkage disequilibrium detected in some localities. ► Individual cattle are infected with a number of genotypes. ► Multiplicity of infection positively correlates with age of host.

## 1 Introduction

Tropical theileriosis is caused by infection with the protozoan parasite *Theileria annulata*. The parasite is transmitted by ticks of the genus *Hyalomma* and causes bovine disease in North Africa, southern Europe, India, the Middle East and Central Asia (Robinson, 1982). The disease can be controlled by the application of acaricides, immunisation with a live attenuated vaccine or chemotherapy (Dolan, 1989; Darghouth et al., 1999). Epidemiological studies have defined two main states of disease: endemic stability where most animals are infected but disease occurs primarily in young calves and endemic instability where challenge is relatively low and disease occurs in animals of all ages (Darghouth et al., 1996). In the bovine host, the parasite replicates asexually, sequentially infecting monocytes and red blood cells but, on ingestion by an infected tick, a morphologically defined sexual stage occurs leading to the production of kinetes (Schein and Friedhoff, 1978), which migrate to the salivary glands to generate the bovine infective sporozoites. Based on cytophotometric measurements of DNA content in the different life-cycle stages of both *T. annulata* and the related parasite *Theileria parva*, it has been concluded that fusion of gametes occurs in the tick gut followed by a two-step meiotic division to yield haploid zygotes that go onto develop into kinetes and sporozoites (Gauer et al., 1995). These results support the conclusion that the parasite is primarily haploid and undergoes genetic exchange during the life-cycle stages in the tick. The occurrence of a sexual cycle has been confirmed in *T. parva* by undertaking crosses between two strains and marker analysis of the progeny (Morzaria et al., 1993). In *T. annulata*, evidence for the occurrence of mating has come from population genetic studies showing the occurrence of random mating (Weir et al., 2007).

Analyses of populations of *T. parva* using micro- and mini-satellite markers have shown a significant multiplicity of infection in field samples and analyses of populations from three regions in Uganda (Oura et al., 2005) and Kenya (Odongo et al., 2006) showed that a high proportion of isolates had unique multi-locus genotypes, thus demonstrating a high level of diversity. Population genetic analyses showed that all of these populations are in linkage disequilibrium (LD) and are therefore not panmictic. Two of the populations in Uganda had an epidemic population structure, where an expansion of a few similar genotypes masked the occurrence of underlying random mating (Oura et al., 2005). The level of diversity in the remaining four populations argues against the parasite largely expanding asexually but the reasons for the observed LD require further investigation. This diversity of population structures is similar to that seen in studies of other apicomplexan species (Beck et al., 2009). For example, it was demonstrated that there are differences in the population structure of *Plasmodium falciparum* in different geographical locations with significant LD observed in six of nine populations studied, corresponding to regions where the transmission rate was low (Anderson et al., 2000). With *T. annulata*, a number of studies have shown that there is significant polymorphism among isolates using isoenzymes (Ben Miled et al., 1994), restriction fragment length polymorphism (RFLP) of single-copy genes (Ben Miled et al., 1994), monoclonal antibodies (Shiels et al., 1986) and sequence analyses of surface antigen genes (Katzer et al., 1994; Gubbels et al., 2000; Schnittger et al., 2002) as well as genes from the schizont secretome (Weir et al., 2010). In a recent study of a Tunisian population, using micro- and mini-satellite markers, linkage equilibrium (LE) was demonstrated leading to the conclusion that there was random mating (Weir et al., 2007). In addition, a small set of isolates from Turkey were genotyped and tentative evidence for geographical sub-structuring was obtained. The Tunisian samples were primarily derived from animals by in vitro cultivation of infected monocytes, raising some concerns about selection in vitro. While this study provided an important advance in our understanding of the population genetics of tropical theileriosis, a number of questions remain to be addressed.

In this study, we analysed a much larger sample set derived directly from blood samples and isolated from a series of geographical locations within both Turkey and Tunisia. Using micro- and mini-satellite genotyping, we addressed the following questions: (i) what is the multiplicity of infection and what variables determine its level? (ii) Is there evidence for geographical sub-structuring and does this occur at the village, region or country level? (iii) Are all populations undergoing random mating or is there evidence for other population structures? (iv) What is the impact of vaccination on parasite diversity? The influence of live, cell line vaccination on the parasite population is currently unknown, both at the level of the individual infected animal and at the level of the local parasite population. Although the immunity induced by cell line vaccination has been shown to be stronger against homologous compared with heterologous parasite challenge (Pipano, 1989), immunisation with a single isolate has proved to be broadly protective in the field. The availability of high-resolution genotyping tools provides us with the capacity to observe the effects of this large-scale immunisation scheme, which carries the potential risk of flooding natural parasite populations with an immunising genotype. This study incorporates sampling sites where vaccination is practised together with sampling sites where it is has not been deployed, and the results provide a comprehensive view of the population structure and diversity of this important pathogen.

## 2 Materials and methods

### 2.1 Parasite material

Eighty-seven bovine blood samples were collected between July 2000 and August 2003, representing primarily clinical cases from two localities in Northern Tunisia – Béja (27 samples) and El Hessiène (44 samples from three farms- Bechir, Hassine and Salah) together with a further 16 samples from Northern Tunisia. Béja is located 100 km from Tunis while El Hessiène is a village located in the Ariana region close to Tunis. Endemic stability is a feature of both areas with respect to tropical theileriosis. All of the samples from Béja were collected from clinically affected adult female cattle in August 2002 during the disease season, while those from El Hessiène were mainly collected from diseased Friesian–Holstein calves between 1 and 6 months of age. Cattle from villages in four districts in Aydın province (Akçaova, Aydın, Incirliova and Nazilli) in Western Turkey were sampled between 1996 and 2003. A total of 96 samples were collected from Akçaova (52 from Sariköy village), 37 from Aydın, 30 from Incirliova and 38 from Nazilli. The town of Aydın lies in the centre of Aydın province with the districts of Akçaova, Incirliova and Nazilli located approximately 40 km south, east and west, respectively. Both calves and adult cattle were sampled, some of which had been vaccinated with a cell line (Teylovac™, Vetal, Turkey). These cattle consisted of mainly dairy types, Holstein and Brown Swiss, together with a small number of an indigenous breed. Seventeen additional samples were analysed from Köşk, Karpuzlu and Kuyucak and, as these locations fell outside the four main sampling districts, they were denoted as ‘other Aydın province’. The locations, numbers of samples and data on the age, breed and vaccination status for the samples from both Tunisia and Turkey are summarised in Table 1. In addition to the field samples, the commercially available Turkish vaccine line Teylovac, derived from the Pendik cell line (Boulter and Hall, 2000), was also genotyped.

This project represents a retrospective analysis of archived material and, consequently, no animal sampling was performed in the course of the study. Permission was sought in both Tunisia and Turkey for the use of the archived sample material.

### 2.2 DNA preparation

EDTA blood samples taken from infected animals were frozen soon after collection and stored at minus 20 °C. Between 100 and 300 μl of whole blood was thawed and the Wizard® Genomic DNA purification system (Promega) was used to prepare DNA according to the manufacturer’s instructions. Genomic DNA was concentrated and desalted by isopropanol precipitation before being dissolved in nuclease-free water and stored at −20 °C. DNA from the vaccine cell line was prepared as previously described (Weir et al., 2007).

### 2.3 Genotyping

The 10 previously described micro-satellite (TS 5, 9, 12 and 16) and mini-satellite markers (TS6, 8, 15, 20, 25 and 31) were used to genotype each DNA sample using PCR amplification with one fluorescently-labelled primer under the conditions described previously (Weir et al., 2007). Amplicons were separated on an ABI 3100 Genetic Analyser in combination with the ROX-labelled GS500 standard size marker set and the Genescan™ data files exported to proprietary software (Genotyper® 3.7., ABI, USA). For each marker, a custom software algorithm was developed in order to define the peaks representing amplicons in each electrophoretogram. The software identified and ‘labelled’ a maximum of 12 peaks within the reference range for each marker, i.e. from a lower limit of between 150 bp and 300 bp depending on the marker, to an upper limit of 500 bp. In order to eliminate minor amplification products, only peaks that were greater than 32% of the maximum peak height were scored and those peaks that were preceded by a higher peak that was within 1.60 bp (+A effect) or were followed by a higher peak within 3.0 bp (stutter) were removed. The program then determined the size and area of the remaining peaks. The predominant peak was defined as that with the largest area under the curve and these predominant alleles for all loci were used to define a multi-locus genotype (MLG) for each sample as well as being used to determine the allele frequency at each locus. To determine the multiplicity of infection, for each sample the total number of alleles at each of the 10 loci was calculated (using the 32% cut-off criterion). For each locus using every sample, the predominant amplicon was ranked in order of ascending size (to two decimal places) to generate a list of allele sizes encompassing the allelic spectrum. Each of these 10 lists was manually examined to allow the creation of fixed bins for the purpose of defining or ‘calling’ actual allele sizes.

### 2.4 Data modelling

Descriptive statistics for the multiplicity of infection (MOI) were calculated and differences in MOIs for the study variable categories were compared using the independent sample *t*-test or the one-way ANOVA using SPSS, v.15 (SPSS Inc., USA). In order to assess the effect of age, vaccination status, breed and gender on MOI, multi-variable regression, using the Generalised Linear Model in SPSS was used. Data from each country was analysed separately. Categorical variables (gender, breed and vaccination status) were entered as fixed factors, while age, the continuous variable, was entered as a co-variate. In the initial model, both the main effects and the two-way interaction terms of the variables were tested. In the final model, only those variables that had significant effects were included. Two-way interactions terms were also omitted because none was significant at *P* ⩽ 0.05. Type III sum of squares (partial sums of squares, where each effect is adjusted for every other effect) were used to test the significance of each fixed effect specified in the model. The adequacy of the fitted models was checked using residual and probability plots.

### 2.5 Population analysis

To investigate patterns of distribution and the underlying trends among parasite genotypes, Principal Component Analysis (PCA) was undertaken. This technique is used to reduce the number of dimensions in a dataset while retaining those characteristics of the dataset that contribute most to its variance. Essentially, a mathematical procedure transforms a number of potentially correlated variables into a reduced number of uncorrelated variables called principal components. The first principal component accounts for as much of the variability in the data as possible, with each successive component accounting for as much of the remaining variability as possible. The Microsoft Excel plug-in ‘Genalex6’ (Peakall and Smouse, 2006) was used to construct a difference matrix and perform PCA on sets of MLG data. The first two axes generated by each analysis were visualised using SigmaPlot 8.0. Similarity comparison of MLGs was undertaken using an allele sharing co-efficient (Bowcock et al., 1994) in Excel Micro-satellite Toolkit (Park, SDE 2001. Trypanotolerance in West African Cattle and the Population Genetic Effects of Selection. PhD thesis, University of Dublin, Ireland). Population genetic analysis to estimate *F*-statistics was performed using the Fstat package version 2.9.3.2. The null hypothesis of LE was tested using LIAN (Haubold and Hudson, 2000), which calculates the standardised index of association $\left(I_{\text{A}}^{\text{S}}\right)$. The software tests for independent assortment of alleles by determining the number of loci at which each pair of MLGs differs and from the distribution of mismatch values, a variance *V*_D_ is calculated which is then compared with the variance expected for LE, termed *V*_e_. The null hypothesis that *V*_D_ = *V*_e_ is tested by either a Monte Carlo simulation or a parametric method and the results provide 95% confidence limits, which are denoted *L*_MC_ and *L*_PARA_, respectively. When *V*_D_ is found to be greater than *L* and a positive value of the index of association is obtained, the null hypothesis is disproved and LD is indicated.

## 3 Results

### 3.1 Moi

The number of alleles at each of the 10 micro- and mini-satellite loci was determined for each isolate. The results showed that every isolate represented a mixed infection, with several alleles identified at one or more loci. The mean number of alleles across all loci was calculated for each isolate to provide a measure of the MOI within each isolate and the data are presented in Table 2. The Tunisian isolates had a mean of 2.51 alleles per locus, whereas Turkish isolates had a mean of 3.15, with both values showing a high SD and a wide range (Table2). Although estimates of the MOI were broadly similar between the two sites of El Hessiène and Béja in Tunisia, site of isolation did exert some influence both at the district level (*P = 0.046*) and village level (*P = 0.031*). Male cattle appeared to have a significantly higher MOI than female (*P = 0.042*). In Turkey, the mean value of MOI varied significantly (*P < 0.001*) ranging from 2.36 in Aydın to 3.68 in Akçaova district. MOI ranged from 1.98 alleles per locus in Sümer Mah village (Nazilli district) to 4.10 alleles per locus in Sariköy village (Akçaova district). There was no significant difference in MOI between the various breeds of cattle (*P* = *0.228*) or sex (*P* = *0.167*). However, vaccinated cattle had a significantly higher MOI than un-vaccinated cattle (*P* < *0.001*). On the basis of these results, the host variables of age, sex, breed, vaccination status and, in Tunisia, the region from which samples were collected, were investigated as to whether they could be used as predictors of MOI in individual animals. Of the 305 isolates from Turkey, 199 had complete data relating to each of these parameters for the host from which they were isolated. These isolates were collected from cattle (61% female) between 3 and 180 months of age, the majority were imported dairy breeds (93%) and one-fifth had a history of cell line vaccination. The effect of age, sex, breed and region on MOI was investigated using a linear regression model (Table 3). Separate models were built for data from each country and for the Turkish population, only isolates from Akçaova were included in the model because the sample sizes from the other regions were too small. Modelling of MOI on the Akçaova data showed that including both vaccination status and breed did not yield any additional information when the effect of the other variable is already known. Therefore, separate models were made for MOI for isolates from Akçaova, one with age and vaccination status and the other with age and breed as explanatory variables (Table 3). All models showed that age, vaccination status and breed of cattle had a significant effect on MOI. When either the effect of breed or vaccination status was taken into account, the MOI increased by a factor of 0.01 for every 1 month increase in age (*P* = *0.016* and *0.034*, respectively). When the effect of age was taken into account, the MOI was higher by 0.63 in vaccinated cattle than in non-vaccinated cattle (*P* = *0.04*). The model also revealed that in Akçaova compared to being a Simmental, being a Brown Swiss was associated with a 1.38 increase in MOI (*p = 0.028*); Holstein–Friesian, 1.56 (*p = 0.012*) and indigenous cattle, 2.20 (*p = 0.002*). While these results appear to contrast with the findings presented in Table 2, when ANOVA was re-performed on the Akçaova dataset alone, breed was revealed to be associated with a difference in MOI (data not shown). Interestingly, the model indicated that when other variables were taken into account, sex is not a significant predictor of infection in Tunisia (Table 3), contrasting with the results presented in Table 2. Therefore, it can be concluded that in both the Turkish and Tunisian populations, the sex of the host is not associated with MOI.

Age was also found to be a significant predictor of MOI in the Tunisian *T. annulata* population (Table 3). After accounting for the effect of other variables, the MOI increased by a factor of 0.11 for every 1 month increase in age (*P = 0.007*). Cattle in the El Hessiène region had a lower MOI by 0.79 compared with cattle from the other northern regions when the effect of other factors was taken into account (*P = 0.012*). Isolates from the Bèja region were not included in the analysis due to missing data. These results strongly support the hypothesis that the number of *T. annulata* genotypes within an individual increases with age, both within the first disease season (i.e. El Hessiène) and during the lifetime of the host.

### 3.2 Population diversity and geographical sub-structuring

**Fig. 1:**
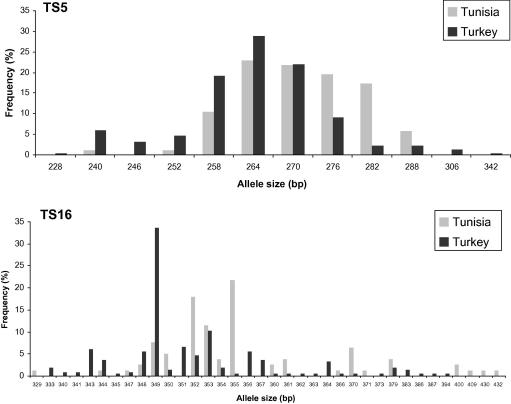
Allele frequencies of TS5 and TS16 in field populations of *Theileria annulata*. The frequency of the predominant allele in each sample was calculated for Tunisian and Turkish *T. annulata* populations and examples of two markers are illustrated. The results of TS5 contrast with TS16 in that the latter shows a large overall number of alleles, a larger proportion of private alleles and generally a greater difference in allele frequency between populations.

**Fig. 2:**
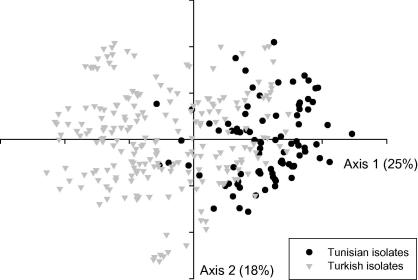
Principal Component Analysis (PCA) of Tunisian and Turkish isolates of *Theileria annulata*. PCA was performed on the multi-locus genotype data representing the Tunisian and Turkish populations of *T. annulata*. The two principal axes generated by this analysis are presented, demonstrating a degree of sub-structuring between isolates from each country. The proportion of the variation in the dataset explained by each axis is indicated in parentheses.

The data generated by genotyping DNA preparations from the Turkish and Tunisian populations were used to construct a MLG for each isolate based on the predominant allele at each locus. A total of 257 MLGs based on 10 loci were defined together with a further 34 MLGs based on nine loci due to the lack of amplification of marker TS9 from some samples. The remaining 14 samples were excluded from the analysis due to a lack of amplification of more than one marker. Based on the automated calling of alleles, the total number of alleles per sample was determined. It is clear from the data that these populations show a high level of diversity. For example there was only one duplicate MLG among the 291 isolates analysed (data not shown) and the number of alleles per sample ranged from one to 12 depending on the locus with the mean ranging from 1.93 for marker TS16 to 3.54 for marker TS8 (Table 4). The diversity within and between the populations from Turkey and Tunisia was examined by determining the number of distinct predominant alleles across the sample set and the data is shown in Table 4, together with an estimate of the gene diversity (heterozygosity) at each locus. In general the heterozygosity is high in both populations ranging from 0.808 (TS15) to 0.967 (TS31) with the exception of the marker TS25, which has values of 0.708 and 0.671 for the Tunisian and Turkish populations, respectively. While there are population-specific alleles, many are common to the two populations and this is illustrated in Fig. 1 for the markers TS5 and TS16. In the case of TS5 there are four alleles only found in the Turkish population and some large differences in allele frequency between the populations (e.g. alleles 276 and 282 bp) while for TS16 there are five Tunisian-specific alleles as well as large differences in allele frequency with other alleles (e.g. alleles 349 and 355 bp). These data suggest that there is some level of geographical sub-structuring between the two populations and to test this, *F*_ST_ values were estimated ($G_{{\text{ST}}^{\prime }}$ and *θ*) between Tunisia and Turkey and the value of 0.052 obtained indicates a moderate degree of differentiation (Table 5). To test whether differentiation could be detected on a smaller geographical scale, populations within each country were analysed. When the Tunisian populations from Béja and El Hessiène were compared, *F*_ST_ was estimated at 0.012, significantly lower than the value of 0.052 measured between the countries, but when the three farms in the village of El Hessiène were compared, $G_{{\text{ST}}^{\prime }}$ was slightly higher at 0.017. This indicated that the amount of differentiation observed between neighbouring sampling sites was of a similar magnitude to that observed when a distance of 100 km separated sampling sites. When the four districts in Western Turkey were compared, $G_{{\text{ST}}^{\prime }}$ was estimated at 0.028. This indicated a higher level of differentiation than observed within Tunisia, but considerably less than that detected between countries. These results support the conclusions that Tunisian and Turkish isolates do not comprise a single population of *T. annulata* and that greater parasite diversity is observed between countries than within them, suggesting a degree of genetic isolation. This differentiation is confirmed by PCA analysis (Fig. 2) where the MLG data from the complete set of samples from Tunisia and Turkey were analysed. The two axes account for 43% of the variation and, while the Turkish and Tunisian populations are not completely distinct, they cluster into different quadrants, again suggesting geographic sub-structuring.

### 3.3 Population genetic analysis

An important question is whether the high levels of diversity observed in these populations can be explained by the frequent occurrence of genetic exchange and whether the evidence for geographical sub-structuring can be strengthened on the basis of evidence for genetic isolation. The MLGs were used to calculate the standard index of association $\left(I_{\text{A}}^{\text{S}}\right)$ to determine whether there is association between alleles when all pair-wise combinations of loci are examined and the values obtained were tested statistically against the null hypothesis of random mating. Combining the Turkish and Tunisian populations or analysing those separately shows that there is LD in both cases (Table 6), although the index of association is low. While there are several possible reasons for LD, we initially investigated the possibility of geographic sub-structuring by analysing populations sub-divided by region and then by village or farm and the results are also presented in Table 6. In Tunisia, LD was observed when the samples from Béja and El Hessiène were combined but when each farm was treated as a single population, LE was detectable within two of the three farms at El Hessiène and within the Béja population as shown in Table 6. The 13 isolates from Hassine farm in El Hessiène displayed a low positive $I_{\text{A}}^{\text{S}}$ value of 0.0500. This indicated a non-random association of alleles, with particular combinations of alleles from different loci found slightly more frequently than would be expected on the basis of random mating. When the Hassine population was removed from the analysis, the rest of the Tunisian dataset reverted to LE, with a low $I_{\text{A}}^{\text{S}}$ value of 0.0059 (data not shown). Consequently, LD was not detected between the Béja and El Hessiène, indicating that the Tunisian population may, in general, be described as panmictic. Similarly, the Turkish populations were sub-divided by district and village and the index of association calculated for each geographically defined population (Table 6). With the exception of Incirliova, $I_{\text{A}}^{\text{S}}$ values above 0.0200 were obtained for each district, indicating the presence of LD in the other three districts. The detection of LE in the Incirliova population demonstrates that there is random mating in this region but geographical sub-structuring between regions in the Turkish population. To further test for this, the populations from the other three districts were divided by village to test for smaller scale sub-structuring. Only those villages with a sample size of 10 of more (Sariköy, Osmanbükü and Sümer Mah) were tested and the results are presented in Table 6. LE was indicated in the village of Osmanbükü in Aydın district, further supporting the existence of geographical sub-structuring and the occurrence of random mating in discrete populations. However, LD was still observed in the villages of Sariköy and Sümer Mah as well as in the districts where these villages are located. These results suggest that in these locations LD cannot be explained simply by geographical sub-structuring. The population from Sariköy includes animals that have been vaccinated and theoretically infection of these animals with the single genotype of the vaccine cell line (data not shown) could distort the population structure. To test this possibility the index of association was recalculated for vaccinates and non-vaccinates separately (Table 6). The vaccinated population showed LD while the un-vaccinated population reverted to LE and this would be expected if the immunising genotype was being detected in a proportion of vaccinated animals (we later tested this concept using similarity analysis). The greatest level of LD was observed in the district of Nazilli and this was, in part, due to 11 samples from the village of Sümer Mah (Table 2), which also showed a low level of MOI (1.98 alleles per locus, Table 2). Consequently, many of these samples shared a high proportion of alleles and this was reflected in the estimated heterozygosity of 0.4891 (Table 6), which was considerably below the range of 0.8–0.9 observed in other villages and districts. When the Sümer Mah samples were removed from the analysis, the Nazilli population remained in LD, although the $I_{\text{A}}^{\text{S}}$ value dropped from 0.1262 to 0.0628 (data not shown). Therefore, in Nazilli district, LD could be detected across all isolates.

### 3.4 Similarity analysis

**Fig. 3:**
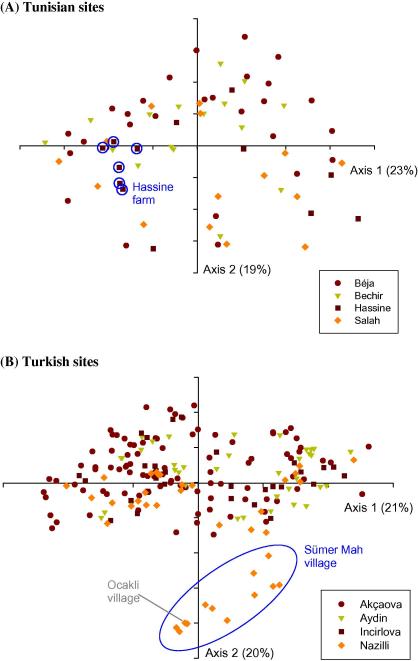
Genotypes of *Theileria annulata* isolated from Tunisia and Turkey with sampling site indicated. Principal Component Analysis (PCA) was performed separately on the multi-locus genotype datasets representing samples from Tunisia and Turkey and the two principal axes generated by each of these analyses are shown. Data points representing isolates are colour-coded to indicate their place of origin. (A) Tunisian sites. The sampling sites in El Hessiène village and Béja are indicated and a cluster corresponding to six isolates from Hassine farm in El Hessiène is highlighted. (B) Turkish sites. The four districts in western Turkey where *T. annulata* were isolated are indicated. A cluster corresponding to 11 isolates from the village of Sümer Mah in Nazilli is highlighted.

MLGs representing isolates from Tunisia and Turkey were analysed separately to assess the similarity among genotypes and to test whether sub-structuring could be detected. Allelic data from all 10 loci were used to construct difference matrices and the results projected onto explanatory axes using PCA. The PCA for the Tunisian isolates was unable to clearly separate isolates from the three farms in El Hessiène (Bechir, Hassine and Salah) and the site at Béja (Fig. 3A). However, it was observed that the majority of the 27 isolates from Béja clustered in the upper two quadrants while most of the isolates from the Salah farm were found in the lower two quadrants. LD was previously identified in the Hassine population and six of the samples clustered together indicating a high degree of relatedness (Fig. 3A), which could account for the LD. When the MLGs were compared directly, four pairs of isolates shared 50% of their alleles. PCA analysis of the genotypes across the four districts in Western Turkey showed that the majority of isolates formed a large cluster across all four quadrants (Fig. 3B) but a proportion of isolates from the district of Akçaova clustered loosely on the leftmost quadrants. One independent cluster was clearly seen, corresponding to all 11 isolates from Sümer Mah village together with a single isolate from Ocakli village in the same district. The population genetic analysis highlighted the Sümer Mah population as having low heterozygosity and a high $I_{\text{A}}^{\text{S}}$ value, and the PCA analysis indicated that these isolates are closely related but highly distinct from the majority of isolates from Western Turkey. These results suggested that there is a degree of sub-structuring which was highlighted by the population genetics analysis. The genotypes from Nazilli district were examined in pair-wise combinations and it was found that 13 isolates showed identity at seven or more loci, further confirming the relatedness of these isolates (data not shown). This raised the question of whether the similarities observed within the Hassine and Sümer Mah populations could be explained on the basis of a clonal complex of strains. To investigate this possibility and determine whether ancestral relationships between the samples could be established, eBurst analysis was performed (Feil et al., 2004). The algorithm attempts to identify the founding genotype within a group of MLGs by identifying single and double locus variant relationships. The genotyped vaccine strain (Teylovac) was included in the analysis, but no founder genotype was identified among the genotypes. Furthermore, similarity analysis with Teylovac showed no similarity with vaccinated strains, suggesting the vaccine is not spreading in the population (data not shown).

The blood samples used to provide parasite material were collected over the space of 2 years in Tunisia and in 1996, 2001 and 2003 in Turkey and thus cover several disease seasons. To investigate whether the time of sampling could explain the distribution of genotypes within each country, the PCA was relabelled to denote the year of origin (data not shown). In the case of both the Tunisian and Turkish samples no specific trend was evident and it can be concluded that the time of sampling did not affect the analyses.

## 4 Discussion

The results of this study indicate that co-infection with multiple genotypes is a common feature of bovine *T. annulata* infection and this agrees with previous work (Ben Miled et al., 1994; Weir et al., 2007). Without isolating single genotypes from a mixed infection it is impossible to determine the exact number of co-infecting genotypes from a sample and therefore the mean number of alleles at each locus is used as a proxy. Although an average of between two and four alleles was identified at each locus in every sample, the actual number of genotypes is likely to be far higher as has been shown in an isolate of *T. parva* where analysis of clonal derivatives revealed 48 distinct genotypes (Katzer et al., 2006).

In the present study, significant regional variation in MOI was identified. In Tunisia, a similar level was observed over all four sampling sites while in Turkey variation between villages was marked and, in general, MOI was higher. For example, the mean number of alleles per locus varied between 1.98 and 4.10 in the villages of Sümer Mah and Sariköy, respectively. Variation between different areas may reflect differences in epidemiology such as transmission intensity, which may be related to variation in the level of tick infestation and/or variation in the prevalence of infection in ticks. In *Plasmodium falciparum*, studies have indicated that the average number of parasite genotypes in a host correlates with the intensity of transmission in an area (Arnot, 1998; Babiker et al., 1999). In Tunisia, the sampling sites were from an area of endemic stability where herds display a limited number of clinical cases during the first disease season with calf sero-prevalence at 100% by autumn after exposure to a high number of a single species of two host ticks (*Hyalomma detritum*) during the summer (Sergent et al., 1945; Flach and Ouhelli, 1992; Flach et al., 1995). Thus one would expect limited variation between sites. In contrast, four species of *Hyalomma* ticks are responsible for the transmission of tropical theileriosis in Turkey (Aktas et al., 2004) and three of those are three-host ticks. The disease season in Turkey is between May and September, with the peak in clinical cases occurring in mid-summer, in parallel with the increase of adult *Hyalomma* tick burdens (Sayin et al., 2003). Further targeted studies are required to determine whether variation in MOI among sampling sites in Turkey is related to the differential distribution of tick species, varying transmission intensities or differing states of endemicity.

A number of host factors may also be involved in determining MOI. For example, it would be predicted that MOI would increase with age due to increased challenge with time or that vaccination, by inducing immunity, would reduce the number of genotypes in an animal. In the Turkish population, vaccinated animals were found to have a statistically significant higher MOI than non-vaccinated animals (3.80 versus 3.00; Table 2, *P < 0.001*). This finding was reinforced by modelling results where a lack of vaccination was found to predict a lower MOI (Table 3). This association is somewhat counterintuitive since it would be expected that vaccine-induced immunity would serve to reduce the number of novel infections. In *P. falciparum* an association has been demonstrated between reduced MOI and acquired anti-malarial immunity (Mayengue et al., 2009). The use of a live attenuated vaccine against *T. annulata* is known to induce the carrier state (Zablotskii, 1991; Singh et al., 2001). However, the genotype similarity analysis excludes the possibility that the immunising strain accounts for an ‘extra genotype’ being identified in vaccinated animals. Two hypotheses could be tested in future studies: (i) the vaccinated cattle are a subset of the population that are in the carrier state whereas the un-vaccinated cattle are predominantly clinical cases with a resultant lower MOI, (ii) cell line vaccination and natural challenge elicit different immune responses and natural immunity is intrinsically associated with a lower MOI. There is evidence that cell-line induced immunity is not wholly protective against heterologous challenge (Pipano, 1981) and this has been linked to strain specificity of the cytotoxic T lymphocyte response (Seitzer and Ahmed, 2008). With the field population of *T. annulata* exhibiting immense diversity, it is difficult to predict how strain-specific immunity operates in the context of the protective response mounted by animals immunised with a clonal cell-line vaccine. Furthermore, with parasite samples collected at a single time-point after vaccination, it is impossible to account for the MOI before immunisation. Consequently, further work is required to determine the factors promoting the apparent increased MOI in vaccinated animals and to discover whether it primarily relates to pre- or post-vaccinal challenge.

The modelling results clearly demonstrate that host age is a significant determinant of MOI (Table 3) in both Tunisia and Turkey. In Tunisia the effect was particularly strong: for every extra month of age, a calf would be expected to harbour a parasite population with an additional 0.11 alleles per locus and this is consistent with multiple re-infections of the animals during the summer season. In *P. falciparum* infection, contrasting results have been found when age was compared with MOI. A positive association was demonstrated with the age of children with clinical malaria in Burkina Faso (Soulama et al., 2009) while a study of asymptomatic children in Senegal concluded that MOI was unrelated to age (Vafa et al., 2008). However, for *T. annulata* our data clearly indicate that the number of co-infecting genotypes gradually builds over time, presumably as the animal is exposed to an array of novel genotypes from ongoing tick challenge.

Our previous study (Weir et al., 2007) concluded that genetic differentiation was detectable between geographically separated populations of *T. annulata* and this has been confirmed using this larger dataset. A moderate level of differentiation between countries was indicated in both studies (*F*_ST_ = 0.049/0.052), which decreased when each country was examined independently. The initial study, which encompassed three diverse sampling areas in Tunisia estimated *F*_ST_ at 0.023, while the new analysis estimated *F*_ST_ at 0.017 between the three contiguous farms at El Hessiène. These results may be interpreted as demonstrating a gradation of genetic differentiation, ranging from a low level found in one sampling site to an intermediate level when sites from different areas in the same country are analysed, to a higher level when samples from different countries are compared. This may be taken as evidence of a degree of genetic isolation between countries consistent with geographical and possibly trade barriers hindering the flow of genetic material. The present study provides a more extensive analysis of diversity in *T. annulata* populations within Turkey, due to the much larger, structured sampling regime. Similar to the initial study, complete genetic isolation between Tunisia and Turkey was not observed (Fig. 2). As the disease is present in countries that lie between Tunisia and Turkey (Libya, Israel, Iran and Iraq), it is possible that while there is no direct cattle movement between Tunisia and Turkey, there is movement between neighbouring countries allowing a limited level of parasite gene flow across the whole region.

The level of genetic differentiation found in the Turkish population when four neighbouring districts were analysed was comparable to that detected within Tunisia and consistent with the initial study, significant LD was detected when Tunisian and Turkish isolates were pooled. This was reduced when the Tunisian collection was analysed on a site-by-site basis however, in contrast to the previous study, a degree of LD was found across Tunisia as a whole. In contrast with the initial study, LD was detected across the entire Turkish population and within three of the four districts sampled in Turkey.

The reasons for LD include a low level of genetic exchange, population sub-structuring, recent immigration, selection and inbreeding (including self-fertilisation). Genetic differentiation and geographical sub-structuring between Tunisian and Turkish populations was consistent with LD being detected at this level. In Tunisia, populations from two farms at El Hessiène and from Béja each displayed LE when analysed independently, with LD indicated only in the Hassine farm. In Turkey isolates from the district of Incirliova and the village of Osmanbükü in Aydın were also in LE, in contrast with the other populations. In these regions it can be inferred there was no impediment to gene flow and the population was truly panmictic, while in areas where LD was observed, some form of restraint on free associations of alleles existed. With LD exhibited in three of the four Turkish districts and population differentiation demonstrated (Table 6), it can be inferred that there was a degree of restriction to gene flow between districts. *Theileria annulata* is maintained in a purely cattle/tick transmission cycle, therefore gene flow between areas must be the result of movement of either the host or the vector, although the tick is likely to migrate over a very restricted range. Logically, cattle would be predicted to be the major vehicle for transporting *T. annulata* genotypes over moderate and large distances. Free movement of cattle over large distances is not a feature in either Tunisia or Turkey, as animals are kept exclusively on farms, although movement of cattle from farm to farm is not an uncommon practice.

The relatively weak LD identified in this study, as indicated by low values of the index of association and the high number of distinct MLGs, are very different from the features of fully clonal bacterial populations (Smith et al., 1993; Haubold et al., 1998). Clearly, a high background level of genetic exchange occurs and the parasite population does not exhibit a clonal structure.

It is possible that when isolates from within a single district are studied, there is micro-geographical sub-structuring whereby herds of cattle are kept in isolation and are infected by isolated populations of ticks. This cannot explain the situation on Hassine farm in Tunisia, where sampled cattle were from closely neighbouring farms. Additionally, the high level of heterozygosity, high MOI and extreme diversity observed in each population would limit the effect of random genetic drift and therefore this is unlikely to explain LD. An alternative explanation is admixture of populations, i.e. the Wahlund effect. This would occur when a distinct parasite population was recently introduced into an area by movement of infected cattle from other localities. It would take a period of time before the pre-existing genotypes and ‘foreign’ genotypes recombined to homogenise the local population and so restore LE. This is highly likely to occur with *T. annulata* as there is only a single round of mating per year in individual ticks. The tick vector is unlikely to travel far in the course of a year, unless major cattle movements occur, and therefore local gene pools are probably stable over the course of a year, with the most significant changes being produced by the introduction of novel genotypes. In Tunisia, it is recognised that cattle are often transported from distant markets and this may explain the situation at Hassine farm. Introduction of foreign genotypes might also be one explanation for the distinct cluster of isolates from the village of Sümer Mah in the Turkish district of Nazilli. As well as being relatively distinct from the majority of the Turkish isolates (Fig. 3B), genotypes were found to be highly related to each other, with a low number of alleles identified at each loci and a low MOI (Table 2). Taken together, these observations suggest a predominant genotype may be sweeping this area.

Perhaps the most likely explanation for LD is the occurrence of self-fertilisation or a level of inbreeding. A considerable number of Turkish isolates share a large proportion of alleles with other isolates from the same district and this indicates a degree of similarity compared with the global polymorphism of each marker. The data indicates a gradation of similarity with genotypes exhibiting varying levels of kinship and this may simply represent related individuals recombining, or it may be associated with self-fertilisation. The fact that only two isolates in the population share a common MLG suggests that self-fertilisation is masked by recombination in ensuing generations. In *P. falciparum*, there is strong evidence of inbreeding and self-fertilisation in natural populations. The structure of *P. falciparum* populations has been shown to be predominantly clonal in regions of low transmission and this has been associated with inbreeding (Anderson et al., 2000) while areas with high transmission intensity exhibit panmixia (Tibayrenc et al., 1990; Anderson et al., 2000; Urdaneta et al., 2001). However, in our study, low MOI in a district, which may indicate low transmission intensity, did not correlate with increased LD. Nevertheless, other studies in *P. falciparum* have indicated that inbreeding may also be a feature of areas of high transmission intensity (Paul et al., 1995; Razakandrainibe et al., 2005) and LD has been identified in two populations from the Republic of the Congo, where transmission is high (Durand et al., 2003). Inbreeding, including self-fertilisation, could certainly explain the LD identified in *T. annulata* populations from localities analysed in this study, although there is no direct evidence for this.

## Figures and Tables

**Table 1:** Host information relating to *Theileria annulata* isolates from Tunisia and Turkey.

Country	*n*	Area	*n*	Age (months)				Sex (*n*)			*n* Vacc
				Min	Max	Mean	SD	M	F	ND	
Tunisia	87	Béja	27	ND	ND	ND	ND	0	27	0	0
		El Hessiène	44	1.5	20.0	5.5	3.3	17	17	10	0
		Bechir	16	3.0	7.0	4.6	1.7	5	6	5	0
		Hassine	13	1.5	20.0	6.1	5.5	5	4	4	0
		Salah	15	2.0	12.0	5.8	2.3	7	7	1	0
		Other Tunisian area (Northern Tunisia)	16	1.0	5.0	3.2	1.0	9	7	0	0

Turkey	218	Akçaova district	96	6.0	180.0	29.3	26.2	54	39	3	32
		Sariköy	52	9.0	180.0	25.4	26.4	42	10	0	28
		Other Akçaova district	44								
		Aydın district	37	8.0	120.0	37.8	25.7	14	23	0	1
		Osmanbükü	12	8.0	84.0	27.7	24.0	4	8	0	1
		Other Aydın district	25								
		Incirliova district	30	3.0	129.0	60.0	34.7	2	24	4	1
		Acarlar	9	24.0	48.0	42.0	12.0	2	3	4	0
		Hao	21	3.0	129.0	63.4	36.6	0	21	0	1
		Nazilli district	38	3.0	138.0	49.8	35.9	6	32	0	6
		Sümer Mah	11	5.0	138.0	49.7	50.3	3	8	0	3
		Kestel	10	3.0	81.0	38.4	25.1	0	10	0	2
		Ocakli	9	33.0	117.0	70.3	30.9	0	9	0	1
		Other Nazilli district	8								
		Other Turkish area (Aydın province)	17	12.0	96.0	30.9	29.8	2	4	11	1

*n *= number of cattle sampled, Min = minimum, Max = maximum, M = male, F = female, ND = no data, *n* vacc = number of cattle which had been vaccinated against tropical theileriosis.

**Table 2:** Summary statistics of multiplicity of infection in samples from Tunisia and Turkey.

Country	Variable	Category	*n*	Number of alleles per locus per isolate				
				Mean	SD	Minimum	Maximum	*P* value
Tunisia	–	*Overall*	87	2.51	0.76	1.25	4.60	–
	Region	Béja	27	2.25	0.64	1.44	3.80	*0.046*
		Unknown	16	2.81	0.76	1.44	4.50	
		El Hessiène	44	2.57	0.78	1.25	4.60	
	Village	Bechir	16	2.26	0.71	1.25	4.10	*0.031*
		Hassine	13	2.92	0.85	1.75	4.60	
		Salah	15	2.58	0.71	1.56	4.20	
		Béja	27	2.25	0.64	1.44	3.80	
	Sex	Male	26	2.73	0.82	1.25	4.60	*0.042*
		Female	51	2.36	0.70	1.30	4.00	

Turkey	–	*Overall*	218	3.15	1.31	1.10	6.11	–
	Region	Akçaova	96	3.68	1.18	1.22	6.11	<*0.001*
		Sariköy	52	4.10	1.02	1.60	6.11	
		Aydın	37	2.36	0.75	1.10	4.50	
		Osmanbükü	12	2.52	0.96	1.20	4.50	
		Incirliova	30	2.69	0.85	1.29	4.40	
		Acarlar	9	2.16	0.57	1.50	3.33	
		Hao	21	2.92	0.85	1.29	4.40	
		Nazilli	38	2.93	0.96	1.30	5.00	
		Sümer Mah	11	1.98	0.29	1.56	2.50	
		Kestel	10	3.26	0.53	2.50	4.00	
		Ocakli	9	4.08	0.55	3.30	5.00	
	Breed	Friesian Holstein	129	3.09	1.07	1.10	5.50	*0.228*
		Indigenous	14	3.57	1.59	1.50	6.11	
		Brown Swiss	49	3.35	1.145	1.22	5.60	
		Simmental	7	2.80	1.41	1.60	5.30	
	Sex	Male	78	3.33	1.297	1.10	6.11	*0.167*
		Female	122	3.09	1.04	1.10	5.40	
	Vaccination status	Vaccinated	41	3.80	1.071	1.10	5.40	<*0.001*
		Un-vaccinated	177	3.00	1.09	1.10	5.40	

*P value* refers to the result of a *t*-test or one-way ANOVA.

**Table 3:** Modelling multiplicity of infection using multi-variable linear regression.

Variable	Co-efficient	SE of coefficient	*P* value	95% Confidence Interval of coefficient	
				Lower	Upper
*Akçaova isolates (age, sex and vaccination status)*					
Intercept	3.82	0.24	<0.001	3.34	4.31
Age	0.01	0.01	0.016	0.002	0.02
Vaccinated = ‘no’^a^	−0.63	0.31	0.04	−1.23	−0.02
Sex = ‘Female’^a^	0.33	0.55	0.551	−0.76	1.42

*Akçaova isolates (age, sex and breed)*					
Intercept	2.04	0.57	0.001	0.91	3.17
Age	0.01	0.01	0.034	0.001	0.02
Breed = ‘Brown Swiss’^b^	1.38	0.62	0.028	0.15	2.60
Breed = ‘Holstein’^b^	1.56	0.61	0.012	0.35	2.77
Breed = ‘Indigenous’^b^	2.20	0.69	0.002	0.83	3.57
Sex = ‘Female’^b^	0.87	0.88	0.329	−0.89	2.63

*Tunisian isolates (age, sex and region)*					
Intercept	2.66	0.27	<0.001	2.11	3.21
Age	0.11	0.04	0.007	0.03	0.19
Sex = ‘Female’^c^	−0.44	0.36	0.231	−1.16	0.29
Region = ‘El Hessiene’^c^	−0.79	0.30	0.012	−1.40	−0.18

SE = standard error.

a Vaccinated = ‘yes’ and sex = ‘male’ are reference categories.

b Sex = ‘male’ and breed = ‘simmental’ are reference categories.

c Sex = ‘male’ and region = ‘unknown’ are reference categories.

**Table 4:** Allelic variation in Tunisian and Turkish populations of *Theileria annulata*.

Criteria		TS5	TS6	TS8	TS9	TS12	TS15	TS16	TS20	TS25	TS31
Number of alleles within each sample	Maximum	8	12	12	8	12	9	6	9	9	10
	Mean	3.03	2.84	3.54	2.96	3.28	3.28	1.93	3.23	2.62	3.00

Number of unique alleles in population	Tunisia	8	28	28	25	33	8	20	21	6	23
	Turkey	12	50	48	32	44	11	30	28	17	54
	Overall	12	61	53	34	49	11	36	34	18	60

Gene diversity (*H*_e_)	Tunisia	0.827	0.946	0.946	0.960	0.956	0.829	0.898	0.885	0.708	0.886
	Turkey	0.818	0.950	0.963	0.947	0.963	0.808	0.858	0.872	0.671	0.967

**Table 5:** Genetic differentiation between field populations of *Theileria annulata*.

Comparison	*n*	*F*_ST_		
		$G_{{\text{ST}}^{\prime }}$	*θ*	*θ* SE
Between Tunisia and Turkey	305	0.052	0.052	0.019

*Tunisia*				
Between El Hessiène and Béja	71	0.012	0.012	0.007
Within El Hessiène (i.e. between Bechir, Hassine & Salah)	44	0.017	0.018	0.012

*Turkey*				
Between Akçaova, Aydın, Incirliova and Nazilli	201	0.028	0.028	0.005

$G_{{\text{ST}}^{\prime }}$ and *θ* are estimators of *F*_ST_ (a measurement of differentiation), SE = standard error.

**Table 6:** Heterozygosity and linkage equilibrium analyses in populations of *Theileria annulata*.

Comparison	*n*	*H*_e_	*H*_e_ SD	No. alleles	No. alleles SD	$I_{\text{A}}^{\text{S}}$	*V*_D_	*L*_para_	*L*_MC_	Linkage
*Tunisia and Turkey*	305	0.902	0.023	36.80	18.81	0.0187	0.9582	0.8351	0.8341	LD
Tunisia	87	0.884	0.025	20.00	9.52	0.0102	1.0599	1.0143	1.0204	LD
El Hessiène & Béja	71	0.883	0.026	18.60	8.68	0.0125	1.0692	1.0111	1.0096	LD
El Hessiène (3 farms)	44	0.872	0.026	14.80	6.00	0.0119	1.1629	1.1414	1.1545	LD
Bechir	16	0.888	0.026	8.70	2.54	−0.0168	0.7798	1.1328	1.1047	LE
Hassine	13	0.844	0.035	6.50	1.72	0.0500	1.6818	1.4518	1.4221	LD
Salah	15	0.848	0.034	7.50	2.37	0.0009	1.2650	1.5603	1.5342	LE
Béja	27	0.887	0.026	11.80	4.52	0.0115	1.0054	1.0198	1.0168	LE
Turkey	218	0.882	0.030	32.60	15.95	0.0197	1.1145	0.9758	0.9769	LD
Akçaova, Aydın, Incirliova and Nazilli	201	0.878	0.031	31.50	15.02	0.0228	1.1659	1.0003	1.0037	LD
Akçaova	96	0.850	0.039	23.30	10.47	0.0252	1.3861	1.2038	1.2084	LD
Sariköy (all samples)	52	0.800	0.046	13.60	5.40	0.0269	1.7139	1.5275	1.5192	LD
Sariköy (un-vaccinated)	24	0.762	0.062	8.50	3.75	0.0175	1.6545	1.7000	1.6909	LE
Sariköy (vaccinated)	28	0.829	0.035	11.10	3.63	0.0349	1.4960	1.4800	1.2795	LD
Aydın	37	0.906	0.023	16.40	5.72	0.0516	1.1513	0.8610	0.8656	LD
Osmanbükü	12	0.889	0.040	8.40	2.27	0.0072	0.8963	1.0828	1.1117	LE
Incirliova	30	0.868	0.033	13.00	5.87	0.0097	1.1111	1.1480	1.1444	LE
Nazilli	38	0.842	0.029	13.00	5.16	0.1262	2.6514	1.3743	1.3893	LD
Sümer Mah	11	0.489	0.069	3.00	1.25	0.0564	3.2209	2.9993	3.1468	LD

*H*_e_ = estimated heterozygosity, no. alleles = mean number of alleles identified across 10 loci. $I_{\text{A}}^{\text{S}}$ = standard index of association, *V*_D_ = mismatch variance (linkage analysis), LD = linkage disequilibrium, LE = linkage equilibrium, *L*_MC_ and *L*_PARA_ = upper 95 % confidence limits of Monte Carlo simulation and parametric tests respectively (linkage analysis). The numbers in bold indicate sub-populations which are in LE and display a low standard index of association.
